# "The Hospital" Medical Book Supplement.—No. XIX

**Published:** 1909-06-19

**Authors:** 


### "THE HOSPITAL" MEDICAL BOOK SUPPLEMENT.—No. XIX.

CONTENTS.

**Medicine—**
A System of Medicine ... ... ... ... ... ... ... 317
Aids to Medicine ... ... ... ... ... ... ... ... 317
**Surgery—**
Appendicitis and Other Diseases of the Vermiform Appendix... 318
A System of Operative Surgery ... ... ... ... 318
Massage in Recent Fractures and other Common Surgical Injuries ... ... ... ... ... ... ... ... ... 318
**Pædiatrics—**
Common Disorders and Diseases of Childhood ... ... ... 319
**Eugenics and Heredity—**
The Scope and Importance to the State of the Science of National Eugenics ... ... ... ... ... ... ... 319
A First Study of the Inheritance of Vision and of the Relative Influence of Heredity and Environment on Sight ... ... 319
**Miscellaneous Items—**
The Natural History of Cancer ... ... ... ... ... ... 320
Health, Morals and Longevity ... ... ... ... ... 320
Chavasse's Advice to a Wife ... ... ... ... ... ... 320
Archives of the Middlesex Hospital ... ... ... ... 320

### MEDICINE.

A SYSTEM OF MEDICINE. Edited by WILLIAM OSLER, M.D., and THOMAS McCRAE, M.D. Vol. V. Oxford Medical Publications. (London. 1909. Pp. 903. 30s. net.)

THIS volume is devoted to diseases of the alimentary tract, and persual of it leaves, on the whole, a sense of insufficiency, of much talk and little substance. This observation does not apply so much to the sections dealing with well-defined diseases of the alimentary system, but rather to the descriptions of the many vague disturbances to which it is liable. The difficulties of the subject and our ignorance of it are pretty well brought out in an introductory chapter by Dr. Stockton, the reading of which, if it does not help one much towards definitive conclusions, at least enables one to appreciate the extraordinary complexity of the problems set by anomalies of digestive function. Perhaps the most noteworthy thing in the opening chapter is the writer's adhesion to the belief that astigmatism and eye-strain are prominent causes of gastric atony. We had thought that this creed had died a natural death, but its resuscitation in an authoritative work reflects the cloudy atmosphere which still surrounds digestive disorders. The chapters upon the diseases of the mouth, and upon those of the œsophagus are satisfactory. As much cannot be said for the chapter upon functional diseases of the stomach, from the pen of Dr. Julius Friedenwald. We freely admit the difficulties of the task to which he was asked to address himself; but the verbiage and barrenness of this chapter are simply monumental. We are introduced to no fewer than twenty-four different varieties of mono-symptomatic gastric neuroses, exclusive of "nervous dyspepsia," which holds the field alone as representing the polysymptomatic group. The author has not invented the terms he uses, but has adopted the classification of Boas. It is a pity he did not leave Boas to gloat alone over his gastro-myxorrhœa, his parorexia, and his gastralgokinesis. This chapter is undoubtedly a blot on the book. The chapter on gastric and duodenal ulcer is better, but much too long, and replete with useless information. In Professor Osler's justly popular text-book upon the principles and practice of medicine the consideration of gastric and duodenal ulcer occupies rather more than eight pages. The corresponding section in this volume runs to close upon fifty pages, and has gained practically nothing by the excess. The total loss to the reader is therefore considerable. The remaining items in this chapter are on the whole satisfactory, though not clearly arranged. The chapter on diseases of the intestines is satisfactory, and that dealing with the peritoneum is good. The latter is written by Dr. H. D. Rolleston, and is much nearer the style of writing which one has a right to expect in an important system of medicine. We may be charged with insular bias, but it is an undoubted relief to escape in this chapter from the windy and redundant writing which characterises the earlier parts of the book. Dr. Eugene Opie's chapter on the diseases of the pancreas is decidedly good, being clear and concise, and the same may be said of Dr. Kelly's chapter upon diseases of the liver and gall-bladder. The book as a whole improves towards the end and contains some really good work. It would be twice as valuable if it were half as long.

AIDS TO MEDICINE. By BERNARD HUDSON, M.D., M.R.C.P. (London: Baillière, Tindall, and Cox. 1909. Pp. 252. Cloth, 3s. net. Paper, 2s. 6d. net.)

THE great objection to all potted text-books is that they aim avowedly and necessarily at being mere examination cram-books. Still, as long as students expect to be crammed for examinations—indeed, demand it—the supply is certain to be met somehow; and from that point of view a good cram-book is better than a bad one. But the education of medical students should be such as to eliminate the desire for cramming, and their examinations should be conducted so as to detect it and reject its devotees. Dr. Hudson's book is very much what one might expect any intelligent student to produce in the process of abstracting his text-books or taking notes of an unusually good course of lectures. We are willing to believe that it is a favourable specimen of its class, but the fact remains that the conditions are a handicap which has been too severe for the author. We can recognise that the necessity for short dogmatic sentences precludes adequate discussion of many points; but surely that is a reason against, rather than in favour of, the reserve which expressed in the statements that "a spirochæte has lately been described" which may be the cause of syphilis, and that sleeping sickness is "probably caused by a trypanosome." Without having examined the work at all exhaustively, we have chanced on many assertions with which we cannot agree. Thus in the first three pages we find enteric fever described as a disease of temperate climates, and its conveyance aerially as commoner than by fomites; the blood omitted in the list of places where the bacilli may be found; and a distinction drawn between enteric fever and typhoid fever, the latter being described as the result of invasion of the lungs or meninges by the specific micro-organism. Worst of all is the unqualified statement that the rash "keeps coming out in crops throughout the whole course of the disease." Among the causes of death of the subjects of tabes dorsalis, ascending urinary sepsis is not mentioned at all, nor is optic atrophy given among the symptoms of disseminate sclerosis. As causes of chlorosis, "copræmia" and "toxic absorption from the intestine due to constipation" are apparently regarded as two different things, and both are discarded in favour of an "explanation" which is a mere empty form of words explaining nothing.

### SURGERY.

Appendicitis and Other Diseases of the Vermiform Appendix By Howard A. Kelly, M.D. (Philadelphia and London : J. B. Lippincott Co. 1909. Pp. 502. Price 25s. net.)

Four years ago Dr. Kelly published, in conjunction with Dr. Hurdon, a monograph upon appendicitis which surpassed everything which has hitherto appeared on this subject, not only in magnitude (for it contained some 820 pages), but in the profusion of its illustrations and their artistic merits, and in the complete and exhaustive consideration allotted to every aspect of this disease. The present new edition has been prepared by Dr. Kelly to meet the daily needs of the great army of general surgeons who yet do not require a work quite so encyclopædic as the original. It is natural, therefore, that the second edition should bear especially on the practical side of the problems which are under consideration, and be curtailed somewhat in other directions.

Throughout the work the difficulties of the practitioner who is confronted with a doubtful case of right-sided abdominal pain are carefully kept in mind, and the indications for treatment, operative and non-operative, are dealt with in an eminently sound and common-sense spirit. Dr. Kelly is totally opposed to the purgative treatment of any patient who may possibly be harbouring an inflamed appendix : his views on this and other points are pithily summed up in nineteen "aphorisms for the general practitioner." There is, we imagine, no point connected directly or indirectly with appendicitis which has escaped Dr. Kelly's notice. Thus he discusses the question whether (1) a healthy or (2) an adherent appendix should be removed when laparotomy is done for some other condition. He pronounces against it in the first case and in favour of it in the second, provided the additional time required does not prejudice the patient's condition. The very complete references to authors and the imposing index of them are valuable features of the book : the most notable omission in this respect is the entire neglect of the work of Head and other British authors on referred pains, and Sherren's areas of hyperæsthesia in this disease. A single, somewhat contemptuous, sentence alluding to a Continental author represents the whole of the information on this subject to be derived from Dr. Kelly's text-book. The illustrations remain as in the first edition, unrivalled.

A System of Operative Surgery. By various authors. Edited by F. F. Burghard, M.S.(Lond.), F.R.C.S. (Eng.), Surgeon to King's College Hospital; Senior Surgeon to the Children's Hospital, Paddington Green. In four volumes. Vol. I. Pp. xxvi+751. (London : Henry Frowde, and Hodder and Stoughton.)

The volume under consideration is the first of four issued by the Oxford Medical Publications, and if the standard set in it is maintained the series should form a valuable addition to medical literature. The volume opens with an instructive and thoughtful article on "The Principles and Technique of Wound Treatment," by Mr. Lockwood. The trend of modern surgical opinion is shown by the assumption that rubber gloves are worn by the surgeon for all operations, an assumption that we are glad to see. The relative merits of asepsis and antisepsis are discussed. A complete account of the methods of local analgesia is supplied by Captain Houghton, R.A.M.C. We are not convinced that there is a wide field of usefulness for this method or that its future is by any means assured; nor do we believe that it is ever likely to displace general anæsthesia as a routine method. But in view of the fact that it is being extensively tried, this detailed account of the technique is especially welcome. The articles on amputations and the operations for the ligature of arteries are from the pen of the chief editor, and we have nothing but admiration for them. The illustrations are particularly good, and a useful innovation, which we do not remember to have seen elsewhere, is the introduction of diagrams showing how an operation ought *not* to be performed. But these two items are in our opinion the dullest part of operative surgery; for as often as not one has to amputate *secundum artem* and not on the lines of a set operation, while the formal operation for the ligature of an artery is quite rarely performed. There is an article on arteriorrhaphy, also by Mr. Burghard, with a special description of endo-aneurysmorrhaphy, also called "Matas' operation." The criticism that we have to make of the procedure is that the operation is difficult and involves a complicated technique, and considering the freedom with which a collateral anastomosis is opened up after ligature of peripheral vessels we hardly consider it necessary. But, as Mr. Burghard points out, "it is in connection with the treatment of aneurysm of the aorta and its main branches that this operation bids fair to be of the greatest importance in the immediate future." There is appended a table of the results of 85 cases of this operation published up to June 1, 1908.

The latter part of the present volume is occupied by a series of articles mainly concerned with the plastic surgery of the nerves, muscles, and tendons, by Mr. Burghard, and one on plastic surgery proper by Mr. Legg. In addition to this, two sections are devoted to the operations for non-tuberculous affections of the bones and of the joints. At first sight this seems curious, but on consideration one appreciates that the surgery of tuberculous bone lesions is so much a separate entity that the editor has been well advised in this respect. As a whole the book gives a frank and lucid description of the manner in which the various operations should be performed and an honest appreciation of their merits, and it is essentially up-to-date. It is not such a mine of information as is Mr. Jacobson's publication on "The Operations of Surgery," but it is much more readable.

Massage in Recent Fractures and other Common Surgical Injuries. By Sir W. H. Bennett, K.C.V.O., F.R.C.S. (London : Longmans, Green and Co. Fourth Edition, 1909. Price, 6s.)

The present edition of a book which ran rapidly through three editions about the beginning of the century contains two new lectures on Sprains and their consequences and on Rigidity of the Spine, while that upon Internal Derangements of the Knee-joint has been omitted; the latter subject the author has more fully discussed in his recent work on injuries and diseases of the knee. In these two chapters the conclusions expressed and the treatment outlined are characterised by the same sound common sense which pervades the original editions. Since the first publication of Sir William's lectures in 1900 the attitude of the profession at large towards injuries of bones and joints has undergone very profound change, partly owing to the influence of his teachings. The procedures he advocates are now no longer unfamiliar, but are widely approved and taught to students by surgeons of special experience in such lesions, and in text-books. At the same time neither the expert nor the novice will find that he has nothing more to learn about massage and movements, and those who have not read Sir W. H. Bennett's monograph may be advised to get the fourth edition and study it at once.

### PÆDIATRICS.

Common Disorders and Diseases of Childhood. By G. F. Still, M.D. (London: Henry Frowde, and Hodder and Stoughton. 1909. Pp. 731, forty figures and charts. Price 15s. net.)

This is another of the Oxford Medical Publications, and, as usual, lacks nothing in style of printing, type, and binding. The author has not been well advised in the choice of a name for his work. The title suggests that only common disorders are included, and that most of these will be considered. Yet a few infrequent diseases are discussed, and many of everyday occurrence absolutely ignored. The most notable omissions are the specific fevers, diphtheria, tonsillar affections, adenoids, whooping-cough, anæmia, purpura, and common skin rashes. Some of these are referred to incidentally in the consideration of other complaints. The style of writing may be described as intermediate between that of a clinical lecture and a textbook. Apparently many of the chapters are clinical addresses modified by the inclusion of a *résumé* and analysis of the author's notes of cases bearing reference to the particular subject. Consequently some of them are very pleasant reading, provided a judicious skipping of the statistical factors is adopted. Unfortunately this style leads to great inequalities in the apportionment of space to matter. Some of the chapters are devoted to diseases, others to symptoms. Those on curd-indigestion, common faults and fallacies in infant feeding, flatulence and colic, indigestion in children past the age of infancy, and bilious attacks, are excellent, in spite of the considerable amount of repetition rendered necessary by this arrangement of the subject-matter. Referring to special points, there are certain omissions, and statements which are insufficiently supported by evidence. Rickets is ascribed to deficiency of fat in the diet, but there is no comment on Findlay's experiments, showing that rickets cannot be produced in animals by diet apart from confinement. Anæmia is stated to be a manifestation of rickets, although there are no special blood changes. Almost certainly it may be due to a diet which causes rickets without being a symptom of it. Much stress is laid on the frequency and importance of "mucous disease," more than most authorities are disposed to admit it deserves. The dietetic treatment of constipation is objected to because it may cause dyspepsia. So it may, in bad cases, but experience shows that simple dietetic measures cure many cases without any recourse to drugs. One is astonished at the scanty space allotted to appendicitis, and one would have liked to have the writer's views on the treatment of hernia by medical measures. Throughout the book considerable space has been devoted to prognosis and treatment. The work will add to the writer's reputation as having a wide experience in the various diseases of children and a thorough knowledge of this branch of medicine. One grave defect, pardonable in a younger writer, is the wearisome reiteration of the egoistical pronoun, hardly compensated for by the apology in the preface for its frequent use, and not rendered forgivable by a generous appreciation of the work of other physicians. Advantageous as the opinion of an individual may be, it carries much more weight with his fellows when it is supported by evidence or the authority of other well-known writers.

---

### EUGENICS AND HEREDITY.

The Scope and Importance to the State of the Science of National Eugenics. By Karl Pearson, F.R.S. Second Edition. (London: Dulau and Co. Pp. 45. Price 1s. net.)

This lecture, which was originally delivered at Oxford as the Robert Boyle lecture in May, 1907, is now re-issued as the first of a series dealing with the problems of eugenics. With the object and aspirations of Professor Pearson we are in cordial sympathy. He endeavours to point out that before any drastic changes are made in the economical administration of the country, it is highly important for statesmen to have a sound knowledge of the fundamental problems of sociology, which, he thinks, can only be obtained by the methods pursued in the Eugenics Laboratory. By numerous "genealogies" and diagrams, Professor Pearson illustrates his views on the inheritance of ability and of diathesis to disease. He considers first the inheritance of physical characters, which can be measured with considerable accuracy. From this he discusses disease, and then passes to an investigation of the inheritance of psychical characters. Lastly, he treats of the question of birth rate and fertility. If we cannot exactly agree with Professor Pearson's philosophical theory of the stages through which a science passes, we nevertheless realise that the work he is doing, as indicated in this volume, should be valuable for a clear comprehension of general tendencies in the progress of the nation. If no answer is given to the question How, facts are at any rate, presented with accuracy and insight, and the conclusions drawn from them are expressed with moderation. If the present workers at the Eugenics Laboratory do not see the fruit of their labours, their carefully collected records will at any rate be useful material for future generations.

A First Study of the Inheritance of Vision and of the Relative Influence of Heredity and Environment on Sight. By Amy Barrington and Karl Pearson, F.R.S. Being No. V. of Eugenics Laboratory Memoirs. (London: Dulau and Co. Pp. 61. Price 4s.)

The authors are happily aware of the real difficulties in the path of the investigator in this field. The difficulty of finding a specialist in ophthalmology who has statistical training, or a statistician who is an eye specialist must, as they admit, detract from the value of this work, as a basis from which general laws may be deduced. Hence this simply a first study. The conclusions at which the authors arrive are the following: (i) No evidence whatever that over-crowded, poverty-stricken homes, or physically ill-conditioned or immoral parentages are *markedly* detrimental to the children's eyesight. (ii) No sufficient or definite evidence that school environment has a deleterious effect on the eyesight of children. (iii) Ample evidence that refraction and keenness of vision are inherited characters, and that the degree of correlation between the eyesight of pairs of relatives is of a wholly different order to the correlation of eyesight with home environment. (iv) Sufficient evidence to show that intelligence as judged by the teacher is correlated with vision in a moderate manner. These conclusions are therefore either tentative or negative, and their value will be realised only when further investigations have been completed. There can, however, be no two opinions about the utility of such work, providing that it is carried out in that spirit of sanity and moderation which has hitherto marked these publications. Professor Pearson is to be congratulated on this pioneer attempt in a very difficult field of research.

### MISCELLANEOUS ITEMS.

The Natural History of Cancer. By W. Roger Williams, F.R.C.S. (London : Heinemann. Pp. 519. Price £1 1s. net.)

When the reader puts down this book he will say to himself "I suspect Mr. Williams is a cynic." If the author was in ironic mood when he offered this work to the medical public, he was a little untender to our deficiencies. So able a man must have been able to see through his own production; he must have said to himself "Let us see how much they will swallow." Here is a monument of industry; a laborious compilation, at once amusing and refreshingly acerbid; a book that might have been both readable and valuable, hopelessly spoiled by a concatenation of statistical pitfalls and dogmatic assertions. An indication of the nicety of some of the propositions he is anxious to prove may be gathered from the following quotation : "From the foregoing considerations, it appears as if predisposition to insanity, like predisposition to tubercle—with which, indeed, it is often allied—gives proclivity to cancer, although developed insanity—like active tuberculous disease — seldom coexists with cancerous lesions." The author betrays such critical acumen in dealing with tables and statistics of others that we suppose it is only the enormous mass of material he has collected which has precluded perfect digestion, and as a consequence permitted several instances of incompatible statements in adjacent paragraphs. One cannot think much of a table of percentages—of ovarian neoplasms, for example—which is classified under the headings "Epithelioma," "Sarcoma," "Non-malignant neoplasms," *and* "Cysts." It would serve no useful purpose to multiply such instances, though it could easily be done. It really is not easy to ascertain exactly what was Mr. Williams' intention, or how he thinks he has advanced the cancer problem. It is well known to every thinking clinician that solution is not to be expected from those who cannot tear their eyes from the top of a microscope; we are all anxious for means of orientation amidst the wide seas of speculation and controversy, and in so far as this book compels the reader to a proper appreciation of perspective it deserves every commendation. But we think the proof-sheets were corrected with a grim smile.

Health, Morals and Longevity. By George Gresswell and Albert Gresswell. Pp. 226. (Bristol : John Wright and Sons. Price 5s. net.)

It is always painful to speak slightingly of an honest effort to advance the sum of human welfare, and this book puts a reviewer in an unpleasant position. For excellence of intention cries aloud from every one of its closely written pages, but, alas ! is out-shouted by inefficiency of execution, which is more vocal still. Much labour has been spent, and the outcome is 226 pages of rambling, discursive platitudinous sermonising. Candour compels the critic to confess that the only part of the book which really interested him is the section devoted to premature burial. Of this accident the authors clearly entertain the most lively apprehension, and one (on the evidence presented) disproportionate to the need. Throwing sense of humour to the winds, they are beguiled into the following expression of opinion : "On the whole, perhaps, the complete severing of the head from the trunk would be the best preventive of live sepulture, bodies being decapitated *before the lid is screwed down.*" The italics are our own. This atmosphere of ingenuousness pervades the whole volume, and disarms the criticism of a kind-hearted person. We feel that the authors are doubtless doing more to advance the happiness of their kind by the example of their lives than they are ever likely to accomplish by their pens. Considering how much better example is than precept, one cannot help regretting that they have strayed into the latter field, for they are not at home in it and are wasting their time there.

Chavasse's Advice to a Wife. Fifteenth edition. Revised by G. Drummond Robinson, M.D., F.R.C.P. (London : J. and A. Churchill. Pp. 360. Price 2s. 6d. net.)

That "Advice to a Wife" has reached the fifteenth edition points to it being a popular book with lay people, to whom no doubt its redundancy, sentimentality, and repetition are attractive rather than the reverse. A great part of it obviously belongs to the days of long ago, when young ladies languished upon sofas, dreaded fresh air, and were ashamed of healthy appetites; whilst young wives considered nourishing stout a necessary accompaniment of pregnancy and nursing. Apparently in those days sherry and other wines to the extent of two glasses per meal were considered by no means superfluous. The introductory chapter (100 pages) is an anachronism, for in these days exercise, fresh air, and an infinitesimal amount of alcohol are the rule rather than the exception among young women. The chapter on pregnancy is clear and contains much useful information, and we note with approval that rest during the daytime is, though somewhat reluctantly, advised as being good for pregnant women. We disagree with the application of spirit to the nipple, and appreciate the more modern suggestion of lanoline or colourless vaseline for the purpose. We are interested to see doubt cast upon the absolute necessity of a binder to preserve the figure. The facts referring to antiseptics, etc., point to the hand of the up-to-date editor, and as they concern the nurse and doctor rather than the patient are treated with a brevity which might be an improvement in other parts of the book. The rest of the work calls for little comment. It consists chiefly of practical common sense, and we can understand it being a comforting, useful, and practical addition to the library of a newly married couple.

Archives of the Middlesex Hospital. Clinical Series No. I. (being the fourteenth volume of the Archives). Edited by W. Sampson Handley and Victor Bonney. (London : Macmillan and Co. 1909.)

This publication constitutes a new departure in the Archives of the Middlesex Hospital, and we look forward to seeing a continuance of this clinical series. In the present issue of 102 pages there are seven papers by members of the staff and four reports by the various registrars. Mr. Pearce Gould contributes four interesting surgical cases; Mr. Bland Sutton gives an account of a case of a parathyroid tumour; Dr. Voelcker contributes a most useful clinical lecture on the significance of dulness in the chest in children. Dr. Wethered describes the graduated labour treatment of pulmonary tuberculosis; Dr. H. Campbell Thompson gives an account of cases illustrating the localisation of cerebral tumours, such as those of the pituitary body with acromegaly, those in the cerebellum, pons, motor cortex, and uncinate gyrus. Mr. Nowell describes some simple effects arising as the result of the presence in the mouth of irritated, dying or dead tooth-pulps; and Mr. Gordon Taylor records a fatal case of perforated gastric ulcer with secondary jejunostomy. All these papers are crisp and short, so that the gist of each is quickly and readily ascertained by the reader.

